# Nano-scale structure and mechanical properties of ASR products under saturated and dry conditions

**DOI:** 10.1038/s41598-020-66262-9

**Published:** 2020-06-08

**Authors:** Huite Wu, Jianwen Pan, Jinting Wang

**Affiliations:** State Key Laboratory of Hydroscience and Engineering, Tsinghua University, Beijing, 100084 China

**Keywords:** Engineering, Nanoscale materials, Composites, Glasses

## Abstract

Alkali-silica reaction (ASR) widely occurs in concrete, resulting in durability problem and premature loss in serviceability of infrastructures all over the world. Understanding of the properties of ASR products, especially at micro scale level, helps mitigation of deterioration in concrete structures. In this study, the nano-scale structure and mechanical properties of the ASR products under dry and saturated conditions are investigated. The scanning electron microscope and energy dispersive spectrometer with X-ray (SEM-EDS) and atomic force microscope (AFM), as well as the nanoindentation are applied to the ASR products. The experimental observation indicates the effect of water on the micromorphology and nano-scale mechanical properties of ASR products. Water makes contribution to the transformation of ASR products from solid to viscous gels and the decrease in the Young’s modulus and hardness of ASR products. The testing results reveal the correlation between nano-scale structure and mechanical properties and improve the understanding of the micromechanical behavior of ASR products.

## Introduction

Alkali-silica reaction (ASR)^1^ is a chemical process where the OH^-^ ions existing in the pore solution attack the amorphous and poorly crystalline silica in aggregates in concrete. Then the generated ASR products absorb water and swell constantly, leading to the increasing stress and fracture of concrete^2^. Understanding the chemical and physical mechanisms of ASR can help the mitigation of ASR. However, current knowledge is insufficient to understand the nano-scale structure and mechanical properties of the ASR products as well as the influence of water, which plays an important role in deterioration of ASR-affected concrete, on the ASR products especially at micro-scale level. Therefore, it is of great significance to study the effect of water on the nano-scale structure and mechanical properties of the ASR products.

With the development of science and technology, more and more advanced microscopic instruments, including scanning electron microscope and energy dispersive spectrometer with X-ray (SEM-EDS) and nanoindentation, are used to investigate the characteristics of the ASR products. SEM-EDS has been demonstrated as an effective way to study the elemental composition and distribution of ASR products. The elemental composition of ASR products is commonly characterized according to the ratio of elements like Ca/Si and Na/Si. Many studies have been made in the mechanism, damage, prevention and microstructure of ASR^2^. Given that recycling the waste glass as aggregate in concrete has the potential risk in the ASR expansion, recent studies focus on the ASR with glass aggregates. For instance, Jun-Ho^3^ used SEM-EDS to study the development of mortar bars with glass aggregate and analyze the different cracks inside the glass particles. They found that the interior cracking was important for the development of the ASR products. Rajabipour^4^ studied the mortar bars with different sizes of glass particles using SEM-EDS and the observation showed that the ASR products came into being in the cracks of glass aggregates instead of the interface between glass and cement. As for nano-scale mechanical properties of ASR products, some researches have been made in recent years. Leemann *et al*.^5^ analyzed the Vickers hardness and E-modulus of the ASR products through micro-indentation tests and the Vickers hardness ranged from 10 to 19, the E-modulus ranged from 7 to 9 GPa, while Zhang *et al*.^6^ made the nanoindentation tests and found that the mean E-modulus was 65 GPa and hardness was 2.5 GPa. These values from different experiments were of great difference, indicating complicated nano-scale mechanical properties of ASR products.

The important role of water on ASR-induced deterioration of concrete is well recognized. Swamy^7^ revealed that water is the necessary for ASR and ASR products swell via water absorption. Larive^8^ placed 4 concrete specimens under different humidity conditions and found an obvious correlation between the humidity and expansion: the higher the humidity, the larger the expansion. Multon^9^ pointed out the water driving influence on ASR expansion and the research revealed that even the water supply is late, the ASR expansion reaches its maximum expansion. Lindgård *et al*.^10^ showed the relation between water uptake and concrete expansion: the porosity increases because of concrete expansion and in turn enhances the water uptake. Yang^11^ prepared recycled glass mortars and blocks based on wet and dry casting methods and compared the ASR in the mortars to study the difference of ASR products between the two kinds of mortars from different production methods. The author found that the ASR products only occurred in the glass aggregates of the mortars. After the chemical reaction, the ASR products absorb free water and swell, then generate tensile stress and induce cracking in the surrounding matrix. The diffusion of ASR gels with absorbing water into the surrounding pores and microcracks depends on the microstructure of the ASR products Besides, the nano-scale mechanical properties of the ASR products affect the cracking of concrete due to ASR products swelling. It would be helpful to understand the ASR-induced deterioration mechanism if the effect of water on the microstructure and nano-scale mechanical properties of ASR products is identified.

This paper focuses on the influence of water on the nano-scale structure and mechanical properties of ASR products. SEM-EDS, atomic force microscope (AFM) and nanoindentation tests are carried out to the recycled glass mortar specimen. Firstly, the work aims to investigate the distribution and elemental composition of the ASR products using SEM-EDS. Then, the relation between the composition in terms of Ca/Si and Na/Si ratio and the location in the crack is built. Lastly, the nano-scale structure and mechanical properties of the ASR products are compared under saturated and dry conditions by the means of AFM and nanoindentation.

## Results

### ASR products under dry and saturated conditions

#### Dry condition

**Figure 1:**
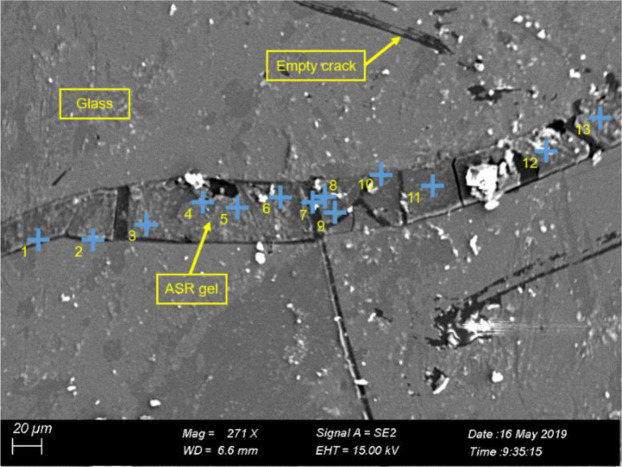
SEM image of a crack filled with ASR gel.

**Figure 2:**
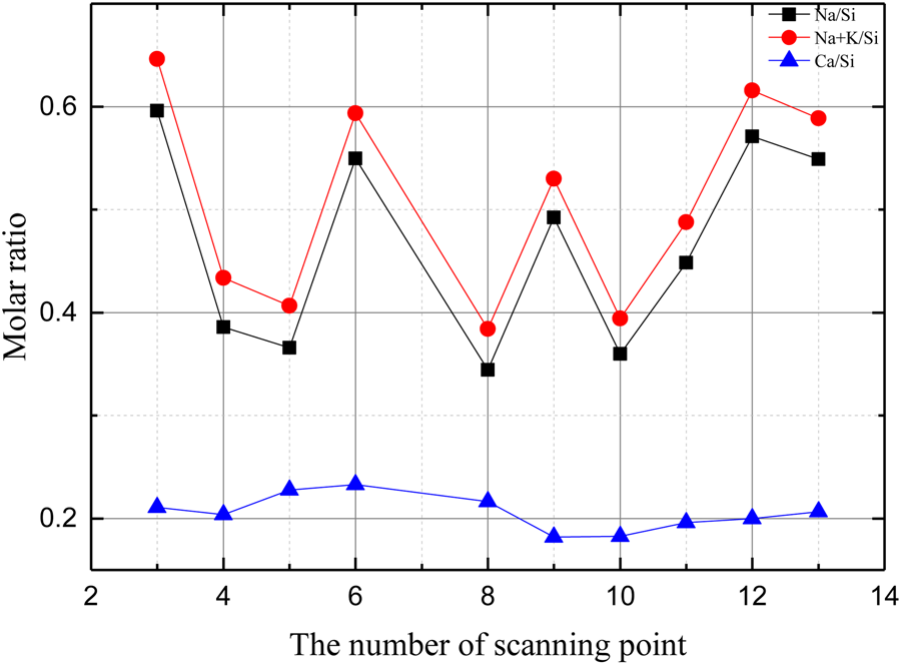
Molar ratios of key elements of the ASR products along the crack in Fig. 1.

The composition and micromorphology of ASR products are studied by SEM-EDS. As is shown in Fig. 1, it is observed that the crack in the glass particle is filled with the ASR products. The elemental composition of the ASR products is tested by EDS in separated testing points along the crack (Fig. 1). Figure 2 shows the variation of Ca/Si, Na/Si and (Na + K)/Na ratios of ASR products with the change of position along the crack. As can be seen from Fig. 2, Ca/Si ratio stays stable along the crack with small fluctuation and ranges from 0.15 to 0.23. Na/Si ratio ranges from 0.27 to 0.60 with fluctuations. (Na + K)/Si ratio ranges from 0.37 to 0.65 and its change tendency is as the same as Na/Si ratio. There are some bright points in the SEM image due to the flawed polishing. In this study, the SEM-EDS experiments are carried out to investigate the distribution and elemental composition of the ASR products. The range of Ca/Si, Na/Si and (Na + K)/Na ratios of the ASR products are consistent with previous research^12^. Thus, from this point of view, there is no significant influence of flawed polish of the SEM samples on the observation of SEM tests.

To avoid the interference with electrons, the samples for SEM-EDS experiments must be completely dry and tested in vacuum environment. Thus SEM-EDS is not capable to investigate ASR products with water. Whereas AFM has been demonstrated as an effective tool to observe ASR products^13^. It allows samples to be saturated and makes it possible to make a comparison of the morphology and roughness of ASR products under two conditions: dry and saturated.

**Figure 3:**
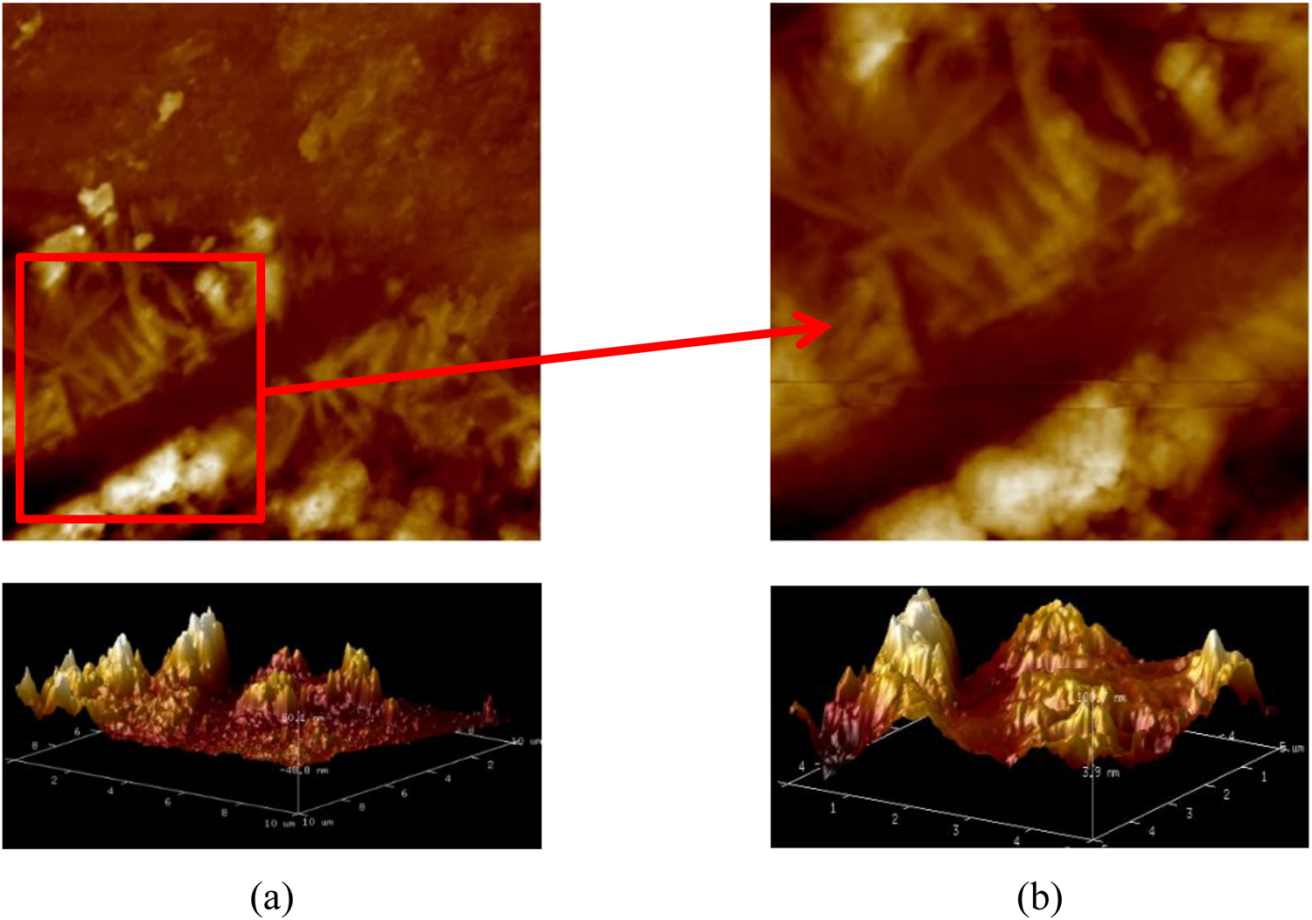
AFM imaging of ASR products at different sizes: (*a*) 10 *μm*; (*b*) 5 *μm*.

Two polished slices are cut from the mortar bars to prepare the samples for AFM tests. The probe scanning area is determined through the optical microscope before the probe begins to scan. Figure 3 shows the photomicrographs of the ASR products captured by AFM at different sizes. In the AFM image, the color represents the height of the corresponding point. The darker color indicates the lower height and the brighter color indicates the higher height. The AFM imaging indicates that the ASR occurs in the glass aggregate. With the development of chemical reaction, the nanoparticles of the ASR products accumulate in the samples leading to the formation of complex feature and latitude difference on the surface. Morphologically, the ASR products distributed in strips or in peaks.

#### Saturated conditions

**Figure 4:**
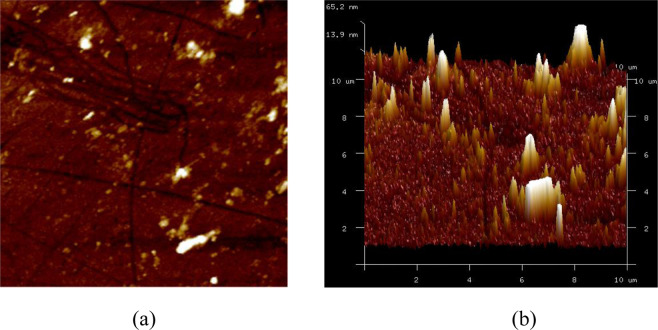
AFM imaging of the glass particle: (*a*) 2D; (*b*) 3D.

The same samples, which are used for AFM investigation under dry condition, are immerged in distilled water until they reach saturation. The same positions of the sample are observed under two different conditions (saturated and dry) to investigate the influence of water on the morphology of ASR products. The surface roughness is described using the Root Mean Square (RMS) of the microscopic peaks and valleys of a surface measured by AFM. The surface roughness of ASR products is used to reflect the degree of undulation. Figure 4 illustrates the two-dimensional (2D) and three-dimensional (3D) AFM images of a glass aggregate. It can be seen that some initial cracks without ASR products and a few nano-protuberances distribute randomly in the glass aggregate. The overall fluctuation of the glass particles is relatively small, and the surface roughness of the glass particles is 11.9 nm.

**Figure 5:**
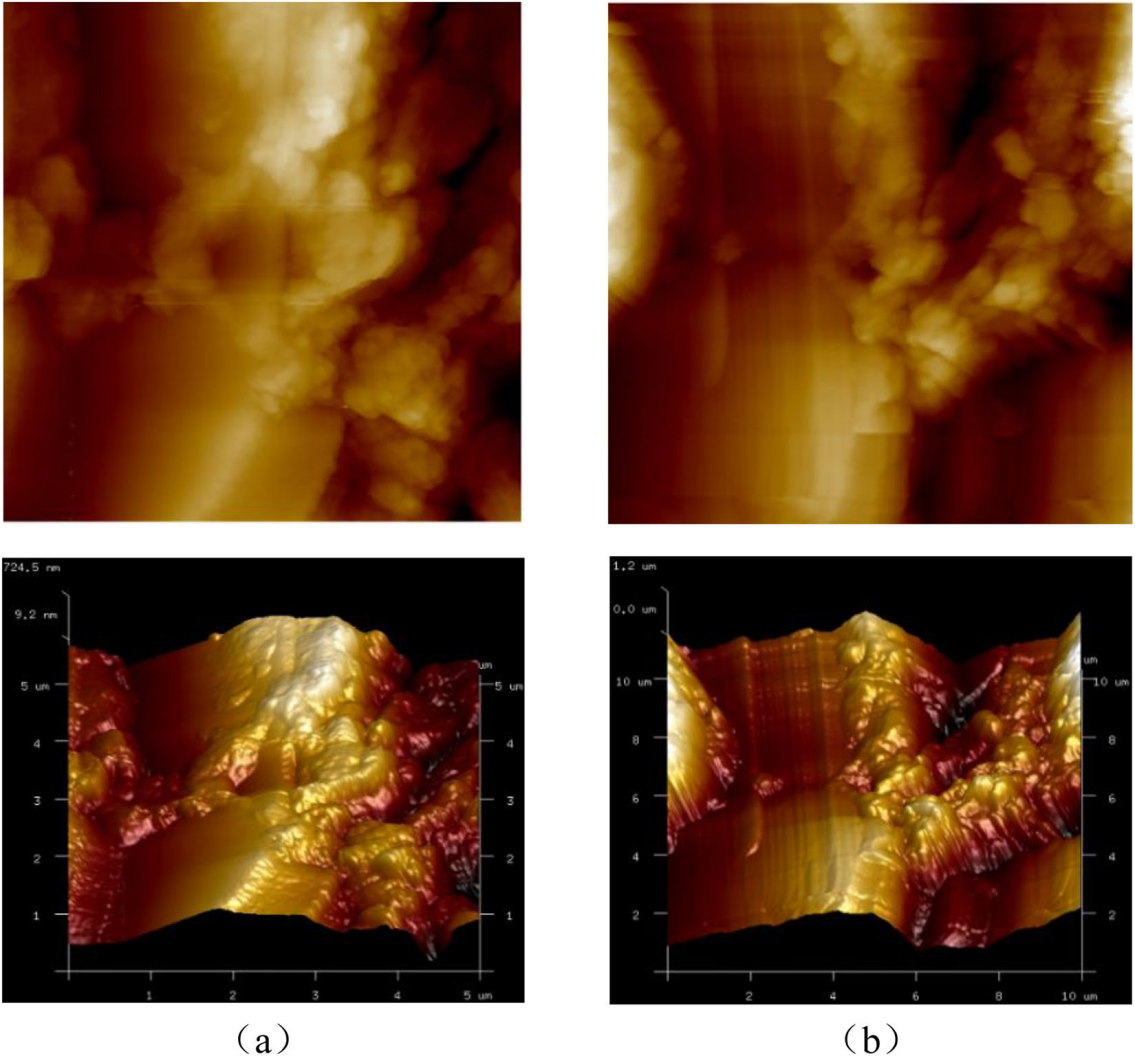
The 2D& 3D AFM images of Sample A under different conditions: (*a*) saturated; (*b*) dry.

**Figure 6:**
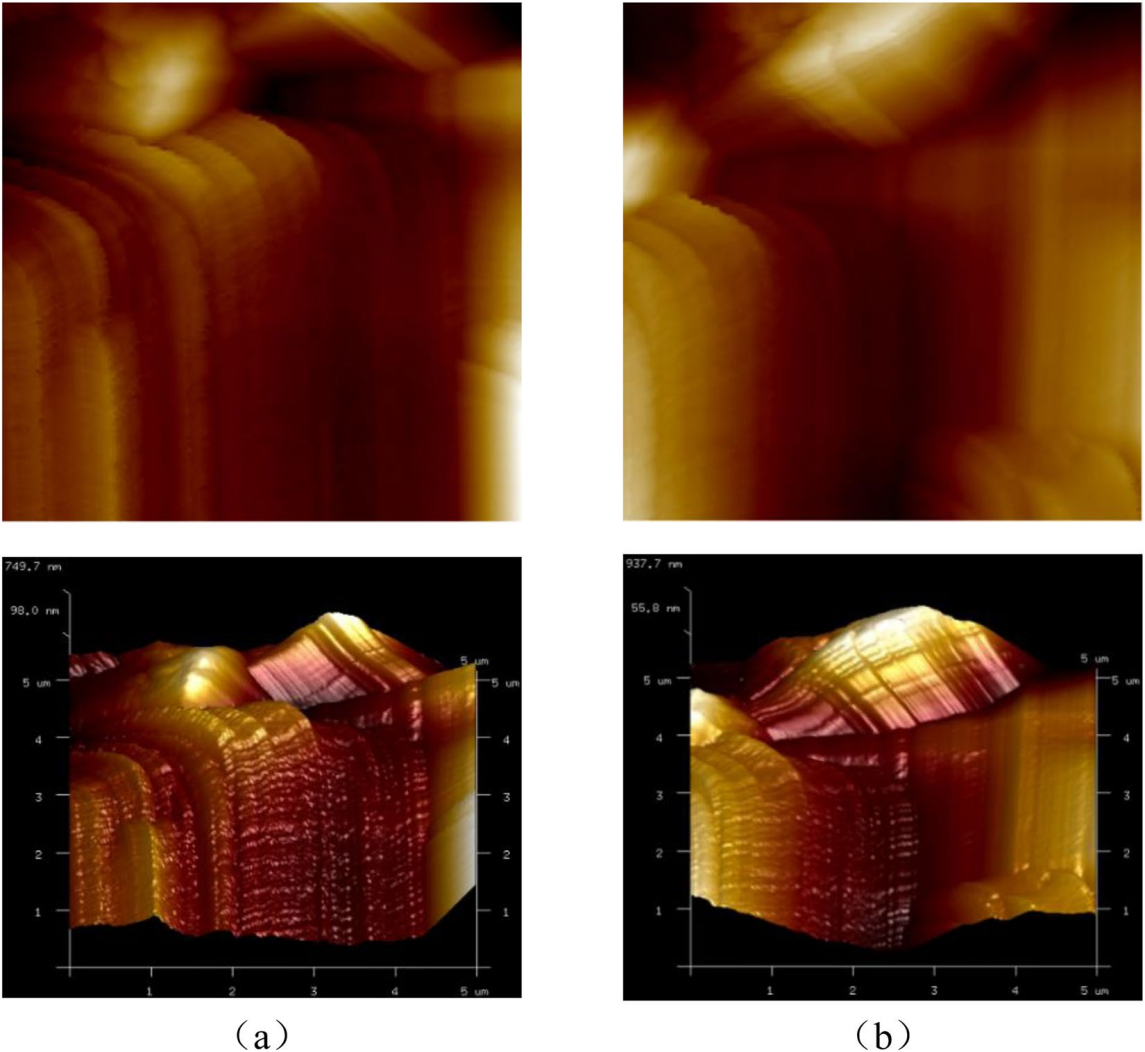
The 2D& 3D AFM images of Sample B under different conditions: (*a*) saturated; (*b*) dry.

Figures 5 and 6 illustrate the AFM images of the ASR products of the two samples, denoted sample A and B, under saturated and dry conditions. As far as the AFM images are concerned, the difference between dry and saturated conditions observed, to the naked eye, is not obvious. Thus, the surface roughness is introduced to evaluate the difference.

The surface roughnesses of the ASR products in sample A are 220 nm and 322 nm corresponding to saturated and dry conditions, respectively, while in sample B are 170 nm and 274 nm. These values are much greater than the surface roughness of the glass particles. In addition, the experimental results show the same variation tendency: the surface roughness of ASR products increases when the ASR products become drier. When under saturated condition, the ASR products swell with water absorption to form the complex morphology. The following disruption may be one explanation of the testing results. The swelling ASR products decrease the porosity which in turn makes the surface of the ASR products flatter and the overall fluctuation less. As the environment becomes drier, there may exist a transformation of the ASR products. The transformation leads to the increase of the solid nanoparticles and these nanoparticles accumulate in the surface, which makes contribution to the complex layered structure. Therefore, it can be seen that the initial accumulation breaks up and more small peaks and valleys appear in Sample A (Fig. 5(b)). On the contrary, the solid nanoparticles accumulate because of the loss of water and higher peaks appear on the surface of sample B (Fig. 6(b)). The evaluation of surface roughness makes it convenient to determine whether the change occurs. However, the detail change about the microstructure of ASR products needs more work to be done.

### Nano-scale mechanical properties of ASR products

**Figure 7:**
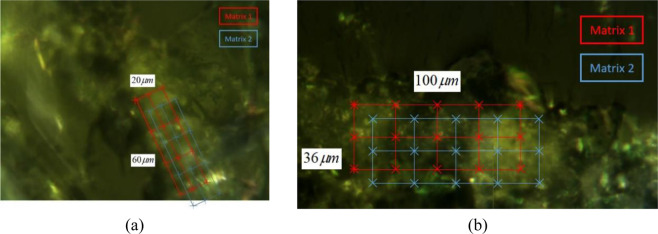
Testing points of the ASR products under different conditions: (*a*) sample A; (*b*) sample B.

Nanoindentation is used to investigate the influence of water on nano-scale mechanical properties of ASR products. Samples A and B, which are used in the AFM tests, are considered. The testing ASR products are at the same positions as the AFM tests. As is shown in Fig. 7, two testing matrixes are designed in two testing areas respectively and the grid points for testing are adopted. Matrix 1 represents the testing points when the Sample is saturated and matrix 2 represents the testing points when the sample is dry. Twelve testing points are investigated for sample A and fifteen testing points for sample B. In this experiment, the depth of the nanoindentation probe at the maximum load is less than 2000nm. Therefore, according to the rule of the nanoindentation, the testing matrixes are designed for samples A and B: 20 *μm* × 60 *μm* and 36 *μm* × 100 *μm*, respectively. The minimum spacing between the indentation points, 10 *μm*, has no negative influence on the nanoindentation results of the testing points.

Given that some testing points may be on the aggregates, the unreasonable data were discarded when performing the statistical analysis. Table 1 shows large variations in the Young’s modulus *E* (5.037–40.029 GPa) and hardness *H* (0.133–2.088 GPa) of the ASR products. The different development of the ASR products leads to the dispersion in nano-scale mechanical properties of the ASR products. And the difference among the testing points indicates that the nanoparticles develop into complex nanostructure with different composition in the aggregate.

**Table 1:** Mix proportion of the mortar bar.

	Cement	Water	Glass aggregate (mm)				
			0.150–0.30	0.30–0.60	0.60–1.18	1.18–2.36	2.36–4.75
Mass (g)	440.0	207.0	148.5	247.5	247.5	247.5	99.0

The average Young’s modulus *E* and hardness *H* of the ASR products under dry condition are 19.115 GPa and 0.540 GPa, respectively, for sample A, and 18.847 GPa and 0.568 GPa for sample B. While the nano-scale mechanical parameters under saturated condition are decreased, *E* = 16.339 GPa and *H* = 0.420 GPa for Sample A, and *E* = 17.287 GPa and *H* = 0.477 GPa for Sample B. The result indicates that the ASR products transform from viscous gels to solid and this transformation causes the increasing in Young’s modulus and hardness with the loss of water, which is consistent with the testing results of AFM.

## Discussion

With the aim to study the elemental composition and morphology of ASR products, SEM-EDX is carried out to make the investigation. In terms of the distribution, the ASR products form in the glass aggregates instead of the interface between the mortars and glass particles. In addition, the ASR products are not continuum, but separated by microcracks. This phenomenon is similar to the observation of the previous studies^4,14–18^. The reason may be that the pore solution flows through the cracks inside the glass aggregates, which gives rise to the accumulation of silica and sodium ions. And this is beneficial for the formation of the ASR products. On the other hand, the cracks limit the expansion of the ASR products inside the aggregates and it is easier for the ASR products to expand along the cracks. Compared with glass aggregate, ASR gel has higher content of calcium. It indicates that calcium is involved in the formation of ASR gel. This phenomenon has been observed previously. The molar ratios of ASR products obtained in this study lies within the typical range^12^, for instance, (Na + K)/Si = 0.1–1.2 and Ca/Si = 0.0–0.2. This experimental result is similar to Liaudat *et al*.^19^, in which the ASR products formed within cracks in glass particles is low in calcium content. The Ca/Si ratio varied from 0.29 to 0.37 and the (Na + K)/Si varied from 0.38 to 0.42.

In order to build a correlation between the nano-scale structure and mechanical properties of the ASR products, the ASR products at the same position of the samples are investigated by AFM and nanoindentation. It can be seen that the variation trend of the surface roughness of ASR products is the same as that of nano-scale mechanical properties from saturated state to dry state. The surface roughness of the ASR products increases from 220 nm to 322 nm for sample A and from 170 nm to 274 nm for sample B when the saturated samples are dried. From the point of view of morphology, the loss of water makes contribution to the transformation of nanoparticles from viscous gel to solid and accumulation of nanoparticles. Complex nanostructure changes the initial surface morphology of the ASR products. The change of the morphology of the ASR products in turn has an influence on their surface roughness. The change of surface roughness of ASR products is not only attributed to the influence of water on ASR products. The immersion of samples in distilled water may cause decomposition of ASR products to some extent, which may interfere the subsequent experiments and analysis. Further studies need to be done about the quantification of the moisture of ASR-gels and the influence of water on the structure of ASR-gels.

On the other hand, the mean Young’s modulus and hardness of the ASR products increased by 17.0% and 28.6% for sample A, 9.02% and 19.08% for sample B with the loss of water. The testing results indicate that there is a strong relationship between the nano-scale structure and mechanical properties. Water has a negative effect on the nano-scale mechanical parameters of the ASR products. When the ASR products are under saturated conditions, the nanoparticles absorb free water and the formation of viscous gel appears resulting in the change of the nano-scale structure of the ASR products. This change leads to the decrease of Young’s modulus and hardness. It is recognized that water plays important role on the deterioration of concrete due to ASR. The ASR products absorb free water and swell, and then produce tensile stress in the activated aggregate. Once the tensile stress exceeds the tensile strength of the aggregate, micro cracks occurs in the aggregate and matrix, leading to deterioration of concrete. Whereas, the decrease in Young’s modulus of ASR products with water reduce the tensile strength as the ASR products swell. It may affect the deterioration mechanism of ASR and further study is needed.

## Conclusions

In this study, the effect of water on nano-scale structure and mechanical properties of ASR products are studied. The SEM-EDS, AFM and nanoindentation tests are carried out. The conclusions can be drawn:

- The elemental composition and micromorphology of ASR products are studied by SEM-EDS. In terms of the distribution, the ASR products occur in the aggregate but instead of the cement pastes or the interfacial transition zones. The ASR products fill with the crack in the glass aggregate. The elemental composition ratios in the ASR products are measured Na/Si ≈ 0.27–0.60 and Ca/Si ≈ 0.15–0.23 and remain stable with small fluctuation along the crack.
- The ASR products under different conditions: saturated and dry are investigated using AFM. The nanoparticles of the ASR products transform from viscous gels to solid because of the loss of water and accumulate into peaks on the surface, which leads to the complex morphology and nanostructure. When the ASR products are dry, the surface roughness of ASR products increases.
- The Young’s modulus *E* and hardness *H* of the ASR products in dry and saturated states are tested using nanoindentation. *E* ranges from 5.037 GPa to 40.029 GPa and *H* is in the range of 0.133 GPa and 2.088 GPa. When the ASR products absorb free water, both the Young’s modulus and hardness decrease, and a greater dispersion is seen in the testing data.
- SEM-EDS and AFM reveal the composition and nanostructure of the ASR products. Additionally, nanoindentation investigates their nano-scale mechanical properties. The combined application of these testing technologies will make contribution to the study in the ASR products at the mirco-scale level and lead to a further understanding in deterioration mechanism of ASR-affected concrete.

## Methods

### Sample preparation

In this study, the experiments according to ASTM C1260 are conducted in order to accelerating alkali silica reaction. PO42.5 cement and the recycled glass are used to prepare the mortar bars with the size of 25 mm × 25 mm × 285 mm. The relative simple glass broken from the abandoned beer bottles, instead of the natural reactive aggregates, is chosen as aggregate to prevent from the influence of other elements on the ASR products. The broken glass particles are firstly washed with water to remove impurities, and then the glass aggregate is put into the oven drying for 24 h before making the mortar bars. The mortar bars are designed on the basis of ASTM C1260^20^. The detail components of mortar bars are shown in Table 2. The mortar bars remain in the molds for 24 h in the moist room before unmolded. After unmolded, the mortar bars are placed in the containers and heated in a water bath at 80 °C for 24 h. Next, the mortar bars are immerged in 80 °C and 1 mol/L NaOH solution for 14 days. After the full reaction, the mortar bars are used to make polished slices for microscopic tests.

**Table 2:** Nano-scale mechanical parameters of the ASR products examined by nanoindentation tests.

Sample	Condition	Property (GPa)	Min	Max	Mean	SD	Number of points
A	Dry	Young’s modulus	8.403	37.187	19.115	9.235	12
		Hardness	0.161	1.156	0.540	0.360	
	Saturated	Young’s modulus	5.037	37.68	16.339	9.127	
		Hardness	0.082	2.087	0.420	0.570	
B	Dry	Young’s modulus	6.221	36.148	18.847	8.973	15
		Hardness	0.035	2.088	0.568	0.536	
	Saturated	Young’s modulus	5.685	40.029	17.287	10.383	
		Hardness	0.104	1.430	0.477	0.453	

Note: SD denotes the standard deviation.

The polished sample with a volume of 12 mm × 12 mm × 5 mm is cut from the middle of the mortar bar and immersed in epoxy for SEM-EDS experiments. The polished sample is sprayed with a 10nm-thick layer of platinum before SEM observations. The samples with a volume of 15 mm × 15 mm × 7 mm for AFM and nanoindentation experiments are only single-side polished instead of impregnated with epoxy to ensure that water can get into the samples. The procedure for the preparation of the samples for microscopic experiments includes cutting, immersion in epoxy, grinding, and polishing^21^.

### SEM Imaging and composition analysis

The polished samples are investigated by SEM-EDS. The distribution and micromorphology of the ASR products are observed by secondary electron (SE2) imaging, and EDS analysis is carried out to characterize the elemental composition of the ASR products. In this study, the operation voltage and working distance are set as 15 kV and 15 mm, respectively.

### AFM Imaging and surface roughness analysis

The Atomic force microscope (AFM) consists of a sharpen probe mounted at the end of a flexible cantilever^22^. AFM is adopted to study the nanostructure and surface roughness of the samples. The comparison of the nanostructure of the ASR products under dry and saturated condition is made through AFM.

### Nanoindentation

**Figure 8:**
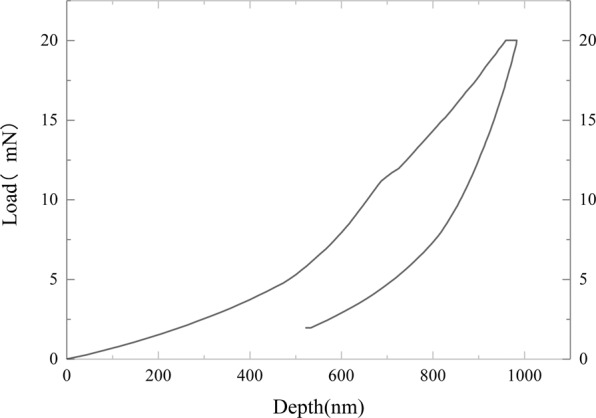
A load-indentation depth curve of a testing point.

As is shown in Fig. 8, a load-indentation depth curve of a testing point presents the nanoindentation process. According to the Eqs. (1) and (2), the Young’s modulus *E* and the hardness *H* of the testing points are calculated from the slope of the unloading process, respectively^23^:

1 $$E={\frac{(1-{\nu }^{2})}{2}\sqrt{\frac{\pi }{A}}\frac{{\rm{d}}P}{{\rm{d}}h}|}_{h={h}_{\max }}$$

2 $$H={\left(\frac{P}{A}\right)|}_{h={h}_{\max }}$$

where $\nu$ means the Poisson’s ratio of the ASR products, which is set as 0.18. And *h*_max_ represents the maximum indentation depth*. A* functions as the contact area between the probe and the ASR products. In this study, the maximum load is 20 mN and maintains for 10 s during the loading process, followed by the unloading process in the indentation tests. The loading and unloading rate are both adopted to be 80 mN/min. The Young’s modulus *E* and hardness *H* of ASR products are measured under dry and saturated conditions.

## Data Availability

The data that support the findings of this study are available from the corresponding author upon reasonable request.
