# Author Correction: Nano-scale structure and mechanical properties of ASR products under saturated and dry conditions

**DOI:** 10.1038/s41598-020-69840-z

**Published:** 2020-07-28

**Authors:** Huite Wu, Jianwen Pan, Jinting Wang

**Affiliations:** State Key Laboratory of Hydroscience and Engineering, Tsinghua University, Beijing, 100084 China

Correction to: *Scientific Reports* 10.1038/s41598-020-66262-9, published online 08 June 2020

This Article contains errors in Table 1 and Table 2. The Tables were inadvertently switched.

The correct Table 1 and Table 2 appear below.

**Table 1:** Nano-scale mechanical parameters of the ASR products examined by nanoindentation tests.

Sample	Condition	Property (GPa)	Min	Max	Mean	SD	Number of points
A	Dry	Young’s modulus	8.403	37.187	19.115	9.235	12
		Hardness	0.161	1.156	0.540	0.360	
	Saturated	Young’s modulus	5.037	37.68	16.339	9.127	
		Hardness	0.082	2.087	0.420	0.570	
B	Dry	Young’s modulus	6.221	36.148	18.847	8.973	15
		Hardness	0.035	2.088	0.568	0.536	
	Saturated	Young’s modulus	5.685	40.029	17.287	10.383	
		Hardness	0.104	1.430	0.477	0.453	

Note: SD denotes the standard deviation.

**Table 2:** Mix proportion of the mortar bar.

	Cement	Water	Glass aggregate (mm)				
			0.150–0.30	0.30–0.60	0.60–1.18	1.18–2.36	2.36–4.75
Mass (g)	440.0	207.0	148.5	247.5	247.5	247.5	99.0

